# Comparison of dosimetries of carbon-ion pencil beam scanning, proton pencil beam scanning and volumetric modulated arc therapy for locally recurrent rectal cancer

**DOI:** 10.1093/jrr/rrac074

**Published:** 2022-11-19

**Authors:** Shinichiro Mori, Tapesh Bhattacharyya, Wataru Furuichi, Naoki Tohyama, Akihiro Nomoto, Makoto Shinoto, Hirotoshi Takiyama, Shigeru Yamada

**Affiliations:** National Institutes for Quantum Science and Technology, Quantum Life and Medical Science Directorate, Institute for Quantum Medical Science, Inage-ku, Chiba 263-8555, Japan; Department of Radiation Oncology, Tata Medical Center, 14, MAR(E-W), DH Block (Newtown), Action Area I, Newtown, Kolkata, West Bengal 700160, India; Accelerator Engineering Corporation, Inage-Ku, Chiba, 263-0043, Japan; Division of Medical Physics, Tokyo Bay Makuhari Clinic for Advanced Imaging, Cancer Screening, and High-Precision Radiotherapy, Mihama-ku, Chiba, 261-0024m Japan; National Institutes for Quantum Science and Technology, QST Hospital, Inage-ku, Chiba 263-8555, Japan; National Institutes for Quantum Science and Technology, QST Hospital, Inage-ku, Chiba 263-8555, Japan; National Institutes for Quantum Science and Technology, QST Hospital, Inage-ku, Chiba 263-8555, Japan; National Institutes for Quantum Science and Technology, QST Hospital, Inage-ku, Chiba 263-8555, Japan

**Keywords:** proton beam, carbon-ion beam, VMAT, dose distribution, radiotherapy, treatment planning

## Abstract

We compared the dose distributions of carbon-ion pencil beam scanning (C-PBS), proton pencil beam scanning (P-PBS) and Volumetric Modulated Arc Therapy (VMAT) for locally recurrent rectal cancer.

The C-PBS treatment planning computed tomography (CT) data sets of 10 locally recurrent rectal cancer cases were randomly selected. Three treatment plans were created using identical prescribed doses. The beam angles for C-PBS and P-PBS were identical. Dosimetry, including the dose received by 95% of the planning target volume (PTV) (D95%), dose to the 2 cc receiving the maximum dose (D2cc), organ at risk (OAR) volume receiving > 15Gy (V15) and > 30Gy (V30), was evaluated. Statistical significance was assessed using the Wilcoxon signed-rank test. Mean PTV-D95% values were > 95% of the volume for P-PBS and C-PBS, whereas that for VMAT was 94.3%. However, PTV-D95% values in P-PBS and VMAT were < 95% in five and two cases, respectively, due to the OAR dose reduction. V30 and V15 to the rectum/intestine for C-PBS (V30 = 4.2 ± 3.2 cc, V15 = 13.8 ± 10.6 cc) and P-PBS (V30 = 7.3 ± 5.6 cc, V15 = 21.3 ± 13.5 cc) were significantly lower than those for VMAT (V30 = 17.1 ± 10.6 cc, V15 = 55.2 ± 28.6 cc). Bladder-V30 values with P-PBS/C-PBS (3.9 ± 4.8 Gy(RBE)/3.0 ± 4.0 Gy(RBE)) were significantly lower than those with VMAT (7.9 ± 8.1 Gy). C-PBS provided superior dose conformation and lower OAR doses compared with P-PBS and VMAT. C-PBS may be the best choice for cases in which VMAT and P-PBS cannot satisfy dose constraints. C-PBS could be another choice for cases in which VMAT and P-PBS cannot satisfy dose constraints, thereby avoiding surgical resection.

## INTRODUCTION

In countries with a higher human development index, rectal cancer is the third most common cause of death and the second most common new malignancy [1]. Therefore, it is crucial to develop new treatments to improve the survival rate of patients with rectal cancer. Surgical resection, chemotherapy and radiotherapy are the main treatment strategies for rectal cancer [2]. Recently, charged particle beam therapies, including those with proton beams [3–5] and carbon-ion beams [6–10], have been applied in rectal cancer treatment. The rates of local recurrence are approximately 5% and 11% for radiotherapy + surgery and surgery only, respectively [11]. Our carbon-ion beam facility has treated more than 13 000 patients since 1994 [12–15]. For locally recurrent rectal cancer treated with carbon-ion beam therapy, the overall survival rates have been 73% at 3 years and 51% at 5 years [16], compared with photon- and proton-beam treatments which had a 3-year overall survival of 64% and 71.3% and 5-year overall survival of 24.9% and 60% (as estimated from figures in [3]), respectively [3,17,18].

In charged particle beam therapy, a passive scattering irradiation technique was initially used in most facilities [19,20]. The pencil-beam scanning (PBS) irradiation technique has been replacing passive scattering, as it provides higher dose conformation [21–23].

New radiotherapy techniques, such as intensity-modulated radiotherapy and volumetric modulated arc therapy (VMAT) that provide improved dose conformation and lower organ at risk (OAR) doses, have been widely introduced [24]. It is of increasing interest to compare these different therapies. Several studies have compared dosimetry between photon and proton beams in different malignancies [25–27], although the number of publications comparing photon, proton and carbon-ion beams is small [28,29]. Since there were evaluated based on the respective treatment protocols, the prescribed dose, beam angle and the number of treatment fractions were different. It is, therefore, a bit hard to understand the effectiveness of these three techniques for locally recurrent rectal cancer; we compared the dosimetry of three techniques for this disease.

## MATERIALS AND METHODS

### Patients and CT acquisition

The imaging data of 15 patients undergoing carbon-ion PBS (C-PBS) treatment were randomly selected from among our previous cases according to the following inclusion criteria: (i) localized recurrence in the form of a pelvic lesion after surgery for primary rectal cancer, (ii) rectal adenocarcinoma or adenosquamous cell carcinoma, (iii) a radiographically measurable tumor < 15 cm, (iv) a performance status < 2, (v) preserved organ function, (vi) no invasion of the digestive tract or bladder, and (vii) no active infection at the tumor site [6]. All patients were informed of the study contents and provided their consent to participate. This study was approved by the institutional review board of our institution (20-038).

All patients were immobilized using a urethane resin cushion (Moldcare®, Alcare, Tokyo, Japan) and low-temperature thermoplastic shells (Shell Fitter®, Kuraray Co., Ltd., Osaka, Japan) [30]. Treatment planning computed tomography (CT) data sets around exhalation were obtained using a 320-detector CT scanner under free breathing condition with a respiratory gating mode (Aquilion One Vision, Canon Medical Systems, Otawara, Japan) [22]. The CT scan conditions were based on our clinical protocols, with a tube voltage of 120 kV and slice thickness of 2.0 mm. The X-ray tube current was adjusted using an automatic exposure control.

### Treatment planning

#### Volume of interest definition

Radiation oncologists manually delineated the target and normal tissue contours (rectum/intestine and bladder) on the treatment planning system (XiO, Elekta AB, Stockholm, Sweden). The clinical target volume (CTV) included a 5-mm margin around the gross tumor volume (GTV) and internal iliac nodes in cases of pelvic sidewall recurrence. Planning target volume (PTV) was delineated by adding a certain margin around the CTV, this margin was depended on the treatment protocol and the treatment modalities due to beam characteristics (penumbra and/or different dose fall-off, etc.). However, we used the universal margin of 5 mm (VMAT/IMRT 5–10 mm [31], proton beam 5–10 mm [32], carbon-ion beam 3–5 mm [10]) for the fair comparison of the three treatment modalities. A 10-mm wide ring-shaped volume of interest (VOI) was inserted 5 mm from the PTV to reduce the OAR dose [33]. Since these VOI definitions were used in our C-PBS treatment protocol, the same VOIs were used for the three treatment modalities. GTV and PTV volumes for respective cases are summarized in Table 1.

**Table 1:** Case characteristics

				Volume (cc)		Beam angle (degrees)	
Case#	Age (y)	Gender		GTV	PTV	VMAT	P-PBS/C-PBS
1	65	M	T2N0M0	21.4	259.8	2 arcs	0, 20, 290, 340
2	52	F	T3N0M0	3.5	60.4	2 arcs	0, 90, 290
3	51	M	T2N2M0	5.7	319.8	2 arcs	20, 70, 90, 340
4	67	F	T3N2M0	6.5	125.3	2 arcs	0, 20, 90
5	60	M	T4N0M0	6.9	122.1	2 arcs	0, 270, 310
6	35	M	T4N2M1	48.5	311.1	2 arcs	0, 90, 270
7	78	M	T3N3M0	5.8	326.8	2 arcs	0, 20, 90
8	77	F	T3N1M0	161.9	376.5	2 arcs	0, 70, 270
9	66	M	T3N1M0	214.8	755.8	2 arcs	0, 70
10	63	M	-	11.8	266.9	2 arcs	0, 20, 90, 270
11	49	M	T3N2M1	2.77	151.02	2 arcs	270, 290, 340
12	49	M	TXN2M0	21.48	261.08	2 arcs	270, 290, 310, 340
13	56	F	T4bN1M0	77.83	323.4	2 arcs	20, 90, 340
14	60	M	-	9.84	461.38	2 arcs	0, 20, 340
15	62	M	T3N3M0	38.62	236.15	2 arcs	105 180 270
Mean	58.3			48.7	292.4		
SD	13.8			75.8	193.4		
Min	35.0			3.5	60.4		
Max	78.0			214.8	755.8		

Abbreviations: VMAT, volumetric modulated arc therapy; PTV, planning target volume; GTV, gross tumor volume; P-PBS, proton pencil beam scanning; C-PBS, carbon-ion pencil beam scanning; SD, standard deviation; Min, minimum; Max, maximum.

#### Dose calculation

To calculate dose distributions for C-PBS, P-PBS and VMAT, the treatment planning system XiO exported structure data and treatment planning information to the treatment planning system (RayStation 6.99; RaySearch Laboratories AB, Stockholm, Sweden). We used dosimetric comparison parameters, including relative biological effectiveness (RBE), which is especially important in C-PBS treatment planning [34] and results in different doses [3–7]. After adjusting the RBE for the particle beams, we used the same prescribed dose for all three treatments (= 73.6 Gy), similar to commonly used treatment protocols [3–9,35,36]. C-PBS and P-PBS treatment planning assumed intensity-modulated particle therapy with subspot dose calculation algorithm [37–40]. The treatment planning parameters (beam spot position and beam spot weight) were optimized to satisfy the clinical criteria (Table 2) using the RBE-weighted absorbed dose [41]. We used the number of beams [2–4] and beam angles used in our C-PBS treatment protocol and set to P-PBS (which usually involves only two beam angles) for C-PBS for accurate comparison (Table 1). The VMAT plan consisted of two full arcs, with the subsegment and the isocenter placed at the center of the PTV using 10 MV beams [42,43]. The collimator angles for each arc were defined on the beam’s eye view graphics as < ± 30°.

**Table 2:** Clinical criteria for the treatment planning

VOI	Dose Metric	Constraint
PTV	D95%	> 95%
GTV	D95%	> 95%
Rectum/intestine	D2 cc	< 46Gy (RBE)
Bladder	D2 cc	< 50 Gy (RBE)
Femoral head	Dmax	< 52 Gy (RBE)

Abbreviations: VOI, volume of interest; PTV, planning target volume; GTV, gross tumor volume; RBE, relative biological effectiveness.

Prescribed doses delivered to the PTV with D50% were 73.6 Gy (corrected for RBE for C-PBS and P-PBS) for all treatments. The number of treatment fractions used was 16. Dose calculation grid is 2 mm for all plans. To improve positional robustness for PTV and GTV, three dimensional robust optimization, allowing for a setup error of 2 mm in all directions, was used to improve dose distributions [44]. Since we assigned higher weight-to-dose constraints to the target than to the OARs, we adjusted optimization objectives (value and weight) to satisfy OAR dose criteria rather than target coverage for respective plans. The optimization objectives (minimum and maximum values) for respective treatment modalities were summarized in Table 3. All treatment plans were performed by the medical physicists, who have clinical experiences for IMRT/VMAT and particle beam treatment planning over 15 years. We compared the dose assessment metrics of the plans using the Wilcoxon signed-rank test with commercial software (MATLAB R2020a®, Mathworks, Natick, MA, USA). We used two-sided *P*-values and *P* < 0.05 was considered statistically significant.

**Table 3:** Optimization objectives for default parameters, and minimum and maximum ranges for respective plans

				Value							Weight						
				Default	VMAT		P-PBS		C-PBS		Default	VMAT		P-PBS		C-PBS	
VOI	Type		Unit		Min	Max	Min	Max	Min	Max		Min	Max	Min	Max	Min	Max
PTV	Dmax		Gy (RBE)	74.34	74.34	74.34	74.34	74.34	74.34	74.34	300	50	600	50	300	50	300
	Dmin		Gy (RBE)	72.86	72.86	72.86	72.86	72.86	72.86	72.86	300	10	1000	300	700	10	800
	Uniform dose		Gy (RBE)	73.6	73.6	73.6	73.6	73.6	73.6	73.6	300	100	1250	200	850	100	300
GTV	Dmax		Gy (RBE)	74.34	73.34	74.34	73.34	74.34	74.34	74.34	300	150	300	300	300	100	300
	Dmin		Gy (RBE)	72.86	72.86	73.86	72.86	73.86	72.86	72.86	300	100	350	20	300	220	300
	Uniform dose		Gy (RBE)	73.6	73.6	73.6	73.6	73.6	73.6	73.6	300	100	300	300	300	300	300
Ring VOI	Dmax		Gy (RBE)	40	35	62.56	30	50	25	62.56	30	3	50	10	100	10	100
Patient - PTV	Dose fall-off low dose distance: 0.5 cm	High	Gy (RBE)	69.92	69.92	73.6	26.22	69.92	59.92	69.92	50	10	100	1	100	1	50
		Low	Gy (RBE)	36.8	29.44	36.8	13.8	36.8	0	36.8							
Rectum /intestine	Dmax		Gy (RBE)	43	30	44	36	46	10	46	100	100	400	100	565	50	350
	V30 Gy (RBE)		cc	20	20	20	20	20	20	20	100	30	100	100	100	30	100
	V15 Gy (RBE)		cc	40	40	40	40	40	40	40	100	30	100	10	100	30	100
Bladder	Dmax		Gy (RBE)	50	30	50	43	50	10	52	100	70	150	50	350	30	400
	V30 Gy (RBE)		cc	10	10	10	10	10	10	10	100	30	100	100	100	30	100
	V15 Gy (RBE)		cc	40	40	40	40	40	40	40	100	30	110	100	100	30	100
Femoral head	Dmax		Gy (RBE)	52	52	52	52	52	52	52	100	10	100	100	100	100	100

Abbreviations: Dmax, maximum dose; Dmin, minimum dose; VOI, volume of interest; PTV, planning target volume; GTV, gross tumor volume; RBE, relative biological effectiveness.

#### Dose assessment

Dose assessments for C-PBS, P-PBS and VMAT were performed using the following metrics: D95% and conformity index (CI) to the GTV and the PTV. CI is calculated by V95/treated volume [45]. The D2cc and the volume of the VOI irradiated with > *n* Gy (RBE) (V*n*) for the rectum/intestine, bladder and the femoral head. We evaluated D2cc because Dmax is the maximum dose to a single voxel and is vulnerable to statistical errors.

**Fig. 1:**
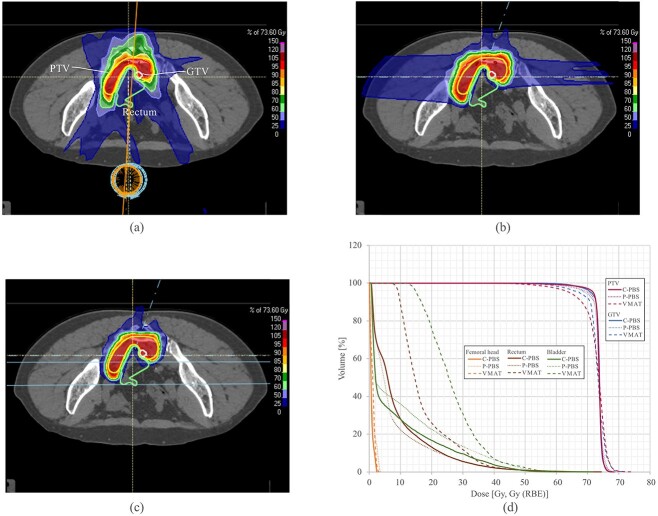
Dose distributions with (a) VMAT, (b) P-PBS, and (c) C-PBS (patient 10). White, yellow, red and light green lines indicate the GTV, PTV and rectum/intestine, respectively. (d) The dose volume histograms for VMAT, P-PBS and C-PBS. *Abbreviations: PTV, planning target volume; GTV, gross tumor volume; RBE, relative biological effectiveness; VMAT, volumetric modulated arc therapy; P-PBS, proton pencil-beam scanning; C-PBS, carbon-ion pencil-beam scanning.*

**Table 4:** Summary of the dosimetry for patient 10

	Dose metric	Unit	VMAT	P-PBS	C-PBS
PTV	D95%	%	87.2	95.6	97.6
	CI		0.9	1.0	1.0
GTV	D95%	%	91.6	94.1	96.6
	CI		0.9	0.9	1.0
Rectum /intestine	D2cc	Gy (RBE)	35.0	30.8	30.2
	V30 Gy (RBE)	cc	4.4	2.1	2.0
	V15 Gy (RBE)	cc	17.2	6.0	7.1
Bladder	D2cc	Gy (RBE)	42.0	42.0	37.2
	V30 Gy (RBE)	cc	11.6	5.0	3.6
	V15 Gy (RBE)	cc	35.5	10.7	8.0
Femoral head	D2cc	Gy (RBE)	36.0	24.5	13.5

Abbreviations: VOI, volume of interest; PTV, planning target volume; GTV, gross tumor volume; CI, conformity index; RBE, relative biological effectiveness; VMAT, volumetric modulated arc therapy; P-PBS, proton pencil beam scanning; C-PBS, carbon-ion pencil beam scanning.

**Fig. 2:**
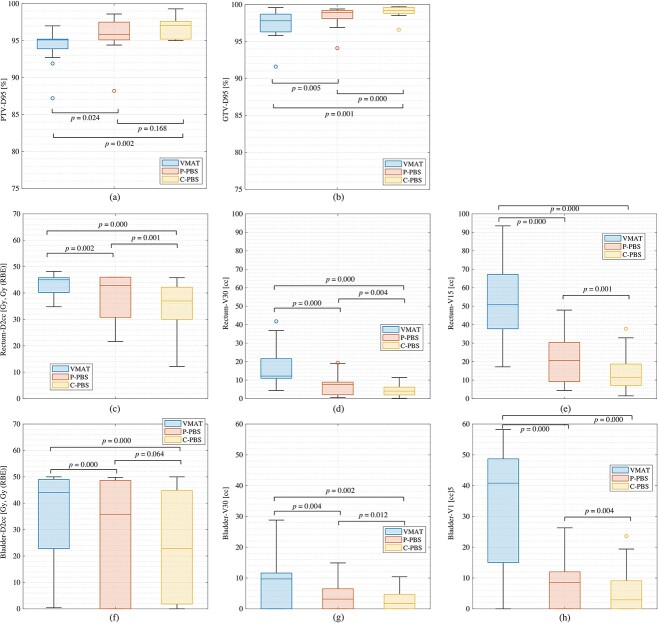
Boxplot of the dose metrics for VMAT, P-PBS and C-PBS for all patients. The central horizontal line in the box indicates the median, and the bottom and top edges of the box indicate the 25^th^ percentiles (*q*1) and 75^th^ percentiles (*q* 3), respectively. The whiskers extend to the most extreme data points not considered outliers. An outlier (marked as circle) is identified if it is > *q*3 + (*q*3 − *q*1) × 1.5 or < *q*1 − (*q*3 − *q*1) × 1.5. *Abbreviations: PTV, planning target volume; GTV, gross tumor volume; RBE, relative biological effectiveness; VMAT, volumetric modulated arc therapy; P-PBS, proton pencil beam scanning; C-PBS, carbon-ion pencil beam scanning.*

## RESULTS

Dose distributions with VMAT for two arcs and P-PBS and C-PBS with a four-field plan (0°, 20°, 90° and 270°) are shown in Figs 1(a)–1(c) (Case 10). This case showed the lowest PTV-D95 with VMAT. Larger 15 Gy (V15) and 30 Gy (V30) regions were observed in the VMAT plan than those in the P-PBS and C-PBS plans. We set a ring-shaped VOI to reduce the doses around the PTV. C-PBS improved PTV dose conformity (CI = 0.97) than P-PBS (CI = 0.95) and VMAT (CI = 0.85). For C-PBS and P-PBS, the V15 and V30 regions between the PTV and rectum/intestine were reduced to satisfy dose constraints. In contrast, the regions covered by 15% and 30% dose with VMAT were slightly larger than those with P-PBS and C-PBS. C-PBS also had a lower dose to the skin and subcutaneous tissues compared with P-PBS and VMAT. Dose-volume histograms for PTV/GTV with VMAT was lower than those with P-PBS and C-PBS (Fig. 1d). Rectal and bladder doses were significantly lower with P-PBS compared with VMAT and were the lowest with C-PBS. The dose assessment metrics (Case 10) are summarized in Table 4. D95% values for the PTV were > 95% for P-PBS and C-PBS, however, those for VMAT was 87.2%. Those for the GTV in C-PBS were 97.6%, however, D95% for P-PBS and VMAT was 94.1% and 91.6%, respectively, likely because the GTV was much closer to the rectum, and minimizing the rectal dose was prioritized over PTV/GTV dose coverage. The D2cc values for the rectum and bladder satisfied the dose constraints. The D2cc, V30 and V15 values for the rectum/bladder decreased in descending order with VMAT, P-PBS and C-PBS.

The dose assessment metrics averaged among all patients for VMAT, P-PBS and C-PBS are summarized in Fig. 2 and Table 5.

**Table 5:** Summary of the dose assessment for each modality averaged over all patients

			VMAT						P-PBS						C-PBS						*P* = value^*^		
VOI	Dose metric	Unit	Mean	±	SD	Min	Max		Mean	±	SD	Min	Max		Mean	±	SD	Min	Max		VMAT /P-PBS	VMAT /C-PBS	P-PBS /C-PBS
PTV	D95%	%	94.3	±	2.3	87.2	97.0		95.8	±	2.5	88.2	98.6		97.0	±	1.5	95.0	99.3		0.024	0.002	0.168
	CI		0.94	±	0.03	0.85	0.97		0.96	±	0.03	0.87	0.99		0.97	±	0.02	0.95	1.00		0.013	0.005	0.331
GTV	D95%	%	97.4	±	2.0	91.6	99.6		98.4	±	1.4	94.1	99.4		99.0	±	0.8	96.6	99.7		0.005	0.000	0.001
	CI		0.99	±	0.02	0.92	1.00		0.99	±	0.02	0.94	1.00		1.00	±	0.01	0.96	1.00		0.041	0.002	0.002
Rectum /intestine	D2cc	Gy (RBE)	43.0	±	4.3	34.8	48.2		39.0	±	8.0	21.6	46.0		34.6	±	9.4	12.2	45.8		0.003	0.002	0.013
	V30 Gy (RBE)	cc	17.1	±	10.6	4.4	41.8		7.3	±	5.6	0.6	19.4		4.2	±	3.2	0.2	11.4		0.000	0.000	0.010
	V15 Gy (RBE)	cc	55.2	±	28.6	17.2	131.4		21.3	±	13.5	4.4	47.9		13.8	±	10.6	1.5	37.8		0.000	0.000	0.013
Bladder	D2cc	Gy (RBE)	32.4	±	19.8	0.4	50.0		24.1	±	22.7	0.0	49.8		21.5	±	21.7	0.0	50.0		0.000	0.000	0.078
	V30 Gy (RBE)	cc	7.9	±	8.1	0.0	28.8		3.9	±	4.8	0.0	14.9		3.0	±	4.0	0.0	10.4		0.004	0.002	0.012
	V15 Gy (RBE)	cc	35.3	±	29.7	0.0	111.6		8.0	±	9.3	0.0	26.3		5.9	±	7.9	0.0	23.6		0.000	0.000	0.004
Femoral head	D2cc	Gy (RBE)	27.9	±	16.5	0.1	48.8		12.8	±	15.3	0.0	40.3		7.8	±	9.6	0.0	31.6		0.000	0.000	0.107

Abbreviations: VOI, volume of interest; PTV, planning target volume; GTV, gross tumor volume; CI, conformity index; VMAT, volumetric modulated arc therapy; P-PBS, proton pencil beam scanning; C-PBS, carbon-ion pencil beam scanning; RBE, relative biological effectiveness; SD, standard deviation; Min, minimum; Max, maximum.

^*^Wilcoxon signed-rank test

For PTV/GTV coverage, the D95% values in C-PBS were > 95% of the prescribed dose in all cases. Mean PTV-D95% values in P-PBS were > 95%; however, two cases had lower D95 values (88.2% and 94.4% for cases 2 and 14, respectively) owing to the OAR dose reduction. PTV-D95% values in VMAT were < 95% in five cases (cases 2, 9, 10, 13 and 14), although mean D95 values with VMAT were 94.3% and 97.4% for PTV and GTV, respectively. PTV CI value in C-PBS (CI = 0.97 ± 0.02) was improved more than that in P-PBS (CI = 0.96 ± 0.03) and VMAT (CI = 0.94 ± 0.03). The box ranges between the 25th and 75th percentiles were lowest with C-PBS, with V-MAT the highest and P-PBS in the middle, resulting in C-PBS satisfying the target coverage requirements for more patients than P-PBS and VMAT (Figs 2a and 2b).

For the rectum/intestine, D2cc values were 43.0 ± 4.3 Gy, 39.0 ± 8.0 Gy (RBE) and 34.6 ± 9.4 Gy (RBE) for VMAT, P-PBS and C-PBS, respectively. Because the PTV was close to the rectum/intestine, the mean D2cc values were similar. P-PBS decreased to the 75th percentile, indicating that P-PBS was associated with a decreased rectal dose, which could not be accomplished using VMAT. C-PBS was improved it rather than P-PBS (Fig. 2c). V30 and V15 values were also reduced in P-PBS and C-PBS compared to those in VMAT (Figs 2d and 2e). These differences were statistically significant (*P* = 0.0001).

The D2cc values of the bladder for P-PBS and C-PBS were not statistically different (*P* = 0.08) (Fig. 2f). The V30 values in P-PBS/C-PBS (3.9 ± 4.8 Gy (RBE)/3.0 ± 4.0 Gy (RBE)) were lower than those in the VMAT group (7.9 ± 8.1 Gy) (*P* < 0.05). However, the V30 values in the P-PBS and C-PBS groups were not different (*P* = 0.01) (Fig. 2g).

The D2cc values of the femoral head for P-PBS (= 12.8 ± 15.3 Gy (RBE)) and C-PBS (= 7.8 ± 9.6 Gy (RBE)) were smaller than that for VMAT (= 27.9 ± 16.5 Gy).

## DISCUSSION

We evaluated the dose distributions in C-PBS, P-PBS and VMAT in locally recurrent rectal cancer. P-PBS and C-PBS significantly reduced the OAR doses compared to VMAT, although the OAR dose limits were satisfied in all plans. C-PBS provided higher PTV dose conformity (CI = 0.97) than P-PBS (CI = 0.96) and VMAT (CI = 0.94).

Because OARs were prioritized over the target, the target coverage for VMAT was lower than that for the other two plans for targets close to OARs. Averaged over all patients, the target coverage for VMAT (PTV-D95% = 94.3%) was lower than those for P-PBS/C-PBS (PTV-D95% = 95.8%/97.0%, *P* = 0.024/0.002). However, the minimum PTV-D95% value for VMAT and P-PBS was 87.2% and 88.2%, respectively. VMAT and P-PBS failed to achieve the target coverage in some cases. C-PBS satisfied all target coverage and OAR dose constraints. This is due to the different beam characteristics as follows. Lateral penumbra for the P-PBS and VMAT are larger than that for the C-PBS. And as the intensity of an X-ray beam is exponentially attenuated as a function of depth. While proton and carbon-ion beams give up most of their energies at the Bragg peak [46], allowing more tailoring of the treatment to hit the target and avoid OARs. In case where the PTV/GTV was close to the OAR, PTV/GTV dose coverage could be degraded to prevent OAR dose constraint. It could be more difficult to satisfy OAR dose constraint, when PTV shape surrounding the OAR (U-shape as shown in Fig. 1).

As increasing the number of beams, the treatment time would be also increased and it might be clinically unacceptable. Albeit that, the number of beams for P-PBS in this study was larger than that generally used in P-PBS, because we used the same number of beams in both C-PBS and P-PBS. The use of a multibeam field achieves a higher dose to the target and a lower dose to surrounding tissues. Dose distribution with P-PBS in this study would be better than that generally used.

VMAT resulted in wider low-dose regions, most likely due to the pursuit of ALARA (as low as reasonably achievement). Our results showed that VMAT satisfied both the target coverage and OAR dose limits in six cases, suggesting that P-PBS or C-PBS would not be necessary.

Given that the construction and operating costs of proton and carbon-ion beam treatment facilities are significantly higher than those of photon beam treatment [47], it may not be advisable to increase the number of treatment centers simply because of the superior beam characteristics of P-PBS and C-PBS. The number of patients could be balanced in each treatment center by selecting the treatment beam type for each patient according to the dosimetry achievable with each modality. As a result, existing treatment centers can improve profitability by increasing the number of patients. Because the number of carbon-ion beam treatment facilities is smaller than that of photon and proton beam centers, long-distance travel might be required. Photon or proton beam treatment at a closer hospital is more convenient.

This study had some limitations. First, the characteristics of photon-, proton- and carbon-ion beams differ in their physical and biological effects. However, we set the same prescribed dose for the respective treatment plans to compare dose distribution differences in this study. A similar study was performed on prostate cancer [48]. They used different prescribed doses of 78 Gy for VMAT and P-PBS and 66 Gy (RBE) for C-PBS based on their treatment protocols. They discussed the relationship between the dosimetry and dose constraints for each plan. However, it is difficult to recognize the dose differences in each treatment beam. Another study of lung treatment using IMRT/VMAT, double-scatter proton beams and C-PBS used the same prescribed dose of 60 Gy (RBE) [29]. However, the prescribed dose can be changed in the future by changing the treatment protocols (e.g. changing the number of fractions and dose escalation studies). However, further studies on dose fractionation are required.

Second, the beam configurations were different for the respective treatment protocols and treatment facilities. P-PBS treatment plans generally use 2–3 beams [3,4]; however, our C-PBS treatment planning protocol routinely uses 2–4 beams. Because comparisons between methods with different numbers of beams would be difficult [49], we set identical beam configurations for the P-PBS and C-PBS.

## CONCLUSION

We compared the dose distributions using three different types of treatment beams. C-PBS provided a superior dose conformation and lower OAR doses than the other beams. Our results showed that C-PBS may be another choice for cases in which VMAT and P-PBS cannot satisfy dose constraints, thereby avoiding surgical resection. P-PBS or VMAT can be used if they satisfy the dose constraints.

## FUNDING

This research was funded by the QST President's Strategic Fund Advanced Study Laboratory.

## CONFLICT OF INTEREST

Dr. Furuichi is employed by/owns an interest in Accelerator Engineering Corporation.
